# Development and evaluation of a cervical cancer-specific restriction spectrum imaging (RSI) model

**DOI:** 10.1016/j.mri.2026.110692

**Published:** 2026-04-16

**Authors:** Ana E. Rodríguez-Soto, Christopher Conlin, Jingjing Zou, Sheida Ebrahimi, Alexandra Besser, Stephan Jordan, Alexandra Schlein, Elin Lundström, Joshua Kuperman, Anders Dale, Tyler Seibert, Michael Hahn, Cynthia S. Santillan, Christopher R. Weil, Gaiane M. Rauch, Jyoti Mayadev, Michael McHale, Rebecca Rakow-Penner

**Affiliations:** a Department of Radiology, University of California San Diego, La Jolla, CA, USA; b Department of Bioengineering, University of California San Diego, La Jolla, CA, USA; c Department of Surgical Sciences, Uppsala University, Uppsala, Sweden; d Center for Medical Imaging, Uppsala University Hospital, Uppsala, Sweden; e J. Craig Venter Institute, La Jolla, CA, USA; f Department of Radiation Medicine and Applied Sciences, University of California San Diego, La Jolla, CA, USA; g Department of Urology, University of California San Diego, La Jolla, CA, USA; h Department of Radiation Oncology, Division of Radiation Oncology, The University of Texas MD Anderson Cancer Center, Houston, TX, USA; i Department of Abdominal Imaging, Division of Diagnostic Imaging, The University of Texas MD Anderson Cancer Center, Houston, TX, USA; j Department of Obstetrics, Gynecology, and Reproductive Sciences, University of California San Diego, La Jolla, CA, USA; k Herbert Wetheim School of Public Health & Human Longevity Science, University of California San Diego, La Jolla, CA, USA

**Keywords:** Cervical cancer, Diffusion-weighted imaging, Restriction spectrum imaging, Tumor conspicuity

## Abstract

**Objective:**

Cervical cancer is the fourth most common cause of cancer and cancer related mortality among women worldwide. MRI is standard of care for cervical cancer staging. The objective of this project is to evaluate the feasibility and performance of an advanced diffusion-weighted imaging (DWI) MRI technique for cervical cancer.

**Methods:**

Forty-four patients with cervical cancer and 22 healthy volunteers underwent 3 T pelvic MRI with reduced field-of-view (FOV) multi-shell diffusion-weighted imaging (DWI; b = 0–3000 s/mm^2^). Patients were divided into model development and independent testing cohorts. Healthy volunteers provided reference distributions for Z-score mapping. RSI models with multi-exponential components were fitted to tumor signals. For each model, the outputs were voxel-wise compartmental signal contributions (C_i,N_) from each water component within tissue. Model performance was assessed using the Bayesian Information Criterion (BIC), tumor contrast-to-noise ratio (CNR), and *Z*-scores. Conventional apparent diffusion coefficient (ADC) was estimated from full-FOV DWI (b = 50–1000 s/mm^2^).

**Results:**

The tetra-exponential model achieved the highest CNR, with only a 6% BIC increase relative to the bi-exponential model. In an independent cohort, CNR was significantly higher in restricted compartments C_1,3_ (median 14.3) and C_1,4_ (median 26.2) compared with ADC. Tumor *Z*-scores were elevated in C_1,3_ (median 3.8) and C_1,4_ (median 8.0), whereas other compartments remained near zero.

**Conclusions:**

RSI with Z-score mapping improved cervical cancer conspicuity compared with ADC, producing interpretable maps that localized tumor signal to restricted diffusion compartments. Based on BIC and CNR, the tetra-exponential RSI model was optimal, supporting RSI as a feasible non-contrast imaging strategy for cervical cancer evaluation.

## 1. Introduction

Cervical cancer is the fourth most common cause of cancer and cancer-related mortality among women worldwide, with over 660,000 new cases and 350,000 deaths in 2022 [1]. Patients with stage ≥ IB2 disease typically undergo radiation or chemoradiation treatment [2], and both fluorodeoxyglucose positron emission tomography/computed tomography (FDG PET/CT) and magnetic resonance imaging (MRI) are recommended by the International Federation of Gynecology and Obstetrics (FIGO) guidelines for staging and treatment planning [3]. FDG PET/CT remains the standard of care for assessing residual or recurrent disease, whereas MRI is widely used for local staging and delineating tumor extent [2,4].

Diffusion-weighted imaging (DWI) is routinely included in pelvic MRI protocols because it improves lesion conspicuity and detection [5]. In radiation oncology, DWI is most often used qualitatively, together with T_2_-weighted imaging and PET/CT, to support staging, tumor detection, and radiotherapy target delineation [6]. On high b-value DWI, cervical tumors typically appear hyperintense relative to background tissues and hypointense on corresponding apparent diffusion coefficient (ADC) maps [7]. These image features reflect how restricted water diffusion associated with higher cellularity in tumor tissues alters the DWI signal [8]. Although ADC has been explored as a quantitative biomarker, its clinical utility remains limited by variability in tumor conspicuity and sensitivity to confounding factors such as edema, necrosis, and inflammation [9,10]. More fundamentally, the mono-exponential ADC model oversimplifies diffusion in tissues [11,12]. In cervical cancer, the DWI signal has been shown to exhibit multi-compartment behavior that is inconsistent with the mono-exponential model [13,14].

Advanced diffusion methods have been developed and applied to cervical cancer to improve tissue characterization beyond the limitations of mono-exponential ADC, particularly in heterogeneous tumors where cellularity, extracellular space, and microvascular flow contribute to the measured signal [13,14]. Intravoxel incoherent motion (IVIM) separates a pseudo-diffusion (perfusion-related) component from tissue diffusivity, while the stretched-exponential model captures deviations from mono-exponential decay associated with tissue heterogeneity [13–15]. However, in the context of cancer imaging, the main tissue feature of interest is restricted diffusion, which reflects increased cellular density and reduced extracellular space [8] and neither IVIM nor stretched-exponential models isolate this component.

Restriction spectrum imaging (RSI) is a diffusion modeling framework that builds on this principle by describing diffusion as the sum of multiple components. Using multi-shell DWI data, RSI separates signal contributions from different water pools within tissues [8]. Because the number of discernible water components is determined by the biophysical properties of the tissue of interest, RSI models are organ-specific [16]. The ADCs of the RSI model compartments are first determined for the tissue of interest and model fitting then determines each compartmenťs contribution to the overall DWI signal. This approach improves tumor conspicuity by isolating restricted diffusion (e.g., tumor) and minimizing other diffusion signal components, including hindered diffusion (edema), free diffusion (e.g., bladder), and flow (e.g., in vessels) [16–18]. RSI has demonstrated improved tumor detection and delineation relative to conventional DWI and ADC. In high-grade brain tumors where it improves delineation of tumor from surrounding edema [16], and in prostate cancer where RSI-derived metrics have been used to improve tumor localization and support radiotherapy planning [18–20].

When RSI is applied voxel-wise and normalized to a healthy reference population, signal contributions associated with restricted diffusion can be converted into standardized *Z*-score maps. These RSI Z-score maps, analogous to previously reported “cellularity maps” [21], increase tumor conspicuity and may facilitate visual and quantitative discrimination of abnormal tissue from normal cervix.

The goal of this study was to adapt RSI to cervical cancer imaging and establish a reliable quantitative MRI technique for cervical cancer assessment. Here, we 1) determined a cervical cancer-specific RSI model, 2) used data from healthy cervical tissue to create *Z*-score maps of RSI-derived measures, and 3) compared tumor conspicuity in RSI-derived measures to conventional ADC.

## 2. Materials and methods

### 2.1. Subjects

Diagnostic MRI was performed in 44 patients with pathology-confirmed cervical cancer between December 2021 and October 2024 under an Institutional Review-Board (IRB) approval. All exams were acquired using a pelvic MRI protocol, that included a multi-shell, high b-value DWI acquisition for lesion conspicuity and target delineation. Disease stage, tumor size, and treatment (i.e., chemoradiotherapy, surgery, etc.) were extracted from their medical records.

Patients were separated into non-overlapping cohorts according to the number of MRI examinations during the study window: a model-development cohort with a single pre-treatment MRI and an independent testing cohort with ≥2 MRI examinations. The primary analysis was to determine the cervix-specific RSI model in the development cohort and evaluate it in the independent cohort using each patienťs baseline pre-treatment examination. Several patients underwent additional pre- or post-treatment MRIs performed for clinical care; because the number and timing of these scans varied widely, they were excluded from the primary analyses. Exploratory analyses of additional examinations evaluated the signal behavior and within-subject variability.

Twenty-two healthy volunteers participated in this IRB-approved study. All healthy volunteers underwent a Papanicolaou smear test within 90 days of MRI. Healthy participants were recruited from physician referrals and via ResearchMatch, a national research volunteer registry created by several academic institutions and supported by the U.S. National Institutes of Health as part of the Clinical Translational Science Award (CTSA) program. ResearchMatch has a large population of volunteers who have consented to be contacted by researchers regarding health studies for which they may be eligible. Written informed consent was obtained from all participants. Inclusion criteria included females weighing less than 136 kg due to scanner table limits. Exclusion criteria included contraindications to MRI (e.g. ferromagnetic implants or devices) and claustrophobia. Age and menopausal status were self-reported.

### 2.2. MRI data collection

Data were initially collected using two 3 T MR750, which were upgraded to SIGNA Premier platforms (DV26.0–30.1, GE Healthcare, Milwaukee, Wisconsin, USA) 14 months into the study, using a 32-channel body array coil. Participants were asked to apply a small amount of sterile gel (Surgilube, HR Pharmaceuticals, Inc., York, PA, USA) intra-vaginally prior to imaging to improve anatomic visualization of the cervix relative to the vaginal vault. Use of the gel was optional, and patients could decline without affecting their participation in the study. The imaging plane of all data relevant to this study was perpendicular to the endocervical canal. The relevant imaging protocol parameters were as follows:

- T_2_-weighted fast spin echo (FSE): TE/TR = 102/7895 msec, flip angle = 111°, field-of-view (FOV) = 200 × 200 mm^2^, acquisition matrix = 300 × 256, reconstructed voxel size = 0.39 × 0.39 × 3 mm^3^.
- Reduced FOV echo-planar imaging (EPI) DW-MRI: TE/TR = 90/5000 msec, b-values (number of diffusion directions) = 0, 50 [6], 250 [6], 800 [6], 2000 [12], and 3000 [12] s/mm^2^, FOV = 100 × 200 mm^2^, acquisition matrix = 32 × 66, reconstruction voxel size = 1.56 × 1.56 × 3.0 mm^3^, 36 slices, spectral attenuated inversion recovery (SPAIR) fat suppression, phase-encoding (PE) direction = anterior-posterior (A/P), and no parallel imaging.
- Full FOV EPI DW-MRI: TE/TR = 60/5000 msec, b-values (number of diffusion directions) = 50 [3], 500 [3], and 1000 [3] s/mm^2^, FOV = 320 × 320 mm^2^, acquisition matrix 128 × 128, reconstruction voxel size = 1.25 × 1.25 × 3.0 mm^3^, 36 slices, SPAIR fat suppression, PE direction A/P, and parallel imaging acceleration factor of 2.

### 2.3. DW-MRI data preprocessing

ReducedFOV DWI datasets were processed using MATLAB 2024b (MathWorks, Natick, MA, USA). Preprocessing included correction for eddy currents [22] and the noise floor [23], followed by the averaging of DWI volumes at each b-value across diffusion directions. Full-FOV clinical DWI data were averaged on the scanner by b-value (three directions per b-value). These images were corrected for the noise floor [23] prior to computing the corresponding ADC maps. Conventional ADC maps were computed in OsiriX MD (version 10.0.2; Pixmeo Sàrl, Geneva, Switzerland) using a diffusion plug-in with a mono-exponential fit.

### 2.4. Region of Interest (ROI) definition

Volumetric regions of interest (ROIs) in the healthy cervixes and tumors were manually drawn on the reduced-FOV b = 800 s/mm^2^ DWI by a medical doctor (S.E.) and reviewed and edited by a fellowship-trained body radiologist (R.R.-P.) on the b = 800 s/mm^2^ DWI images. Anatomical T_2_-weighted images were used to guide ROI placement. In patients, ROIs excluded macroscopic necrosis, cysts and hemorrhage or susceptibility artifacts. To reduce partial-volume effects, the ROIs were morphologically eroded by one voxel in 3D space using MATLAB imerode function with a cubic structuring element. These ROIs were used for all reduced-FOV DWI analyses.

For conventional ADC measurements from full-FOV DWI, the eroded tumor ROIs (in reduced-FOV DWI space) were mapped to the full-FOV DWI data using a patient-space transform in left-posterior-head (LPH) coordinates and resampled with cubic interpolation. After resampling, the masks were binarized again by thresholding at 0.6 (values <0.6 set to 0; values ≥0.6 set to 1).

### 2.5. RSI modeling

To determine cervical cancer-specific RSI models, multi-exponential functions were fitted to voxel-wise diffusion-weighted signals from tumor ROIs [16–18]. Global compartment diffusivities (D_i,N_) were first estimated across all tumor voxels, and fixed diffusivity values were then estimated for each model order (*N* = 2, 3, and 4). Signal contributions (C_i,N_) were then estimated voxel-wise as non-negative values using these fixed D_i,N_. This approach allows the use of a consistent model across all voxels to compare signal contributions across water compartments.

The diffusion signal was modeled as the linear combination of N exponential decays:

(1) $$S_{\text{diff}}\;(b,N)=S_{o}\sum_{i}^{N}e^{-bD_{i,N}}=\sum_{i}^{N}C_{i,N}e^{-bD_{i,N}}$$

where C_i,N_ is the signal contribution (arbitrary units) of each exponential component and D_i,N_ is the diffusivity of the i^th^ exponential component (ordered D_1,N_ < D_i,N_; D_1,N_ denotes the slowest component). Variable b represents the diffusion weighting in s/mm^2^. Fixed D_i,N_ values were estimated using 5-fold cross-validation across tumor voxels from the RSI model development cohort, and the median values across the folds were used to define the final model parameters. Fits were performed on noise floor-corrected magnitude data with monotonic ordering constraints on D_i,N_.

The Bayesian information criterion (BIC) was calculated for each model order as a measure of goodness of fit relative to model complexity, explicitly penalizing models with more parameters to prevent overfitting. A lower BIC value indicates improved model fit. All BIC values were normalized relative to the lowest BIC value (i.e., the best-performing model), providing a relative measure of model fit (ΔBIC).

### 2.6. Tumor conspicuity

Tumor conspicuity was quantified by measuring the contrast-to-noise ratio (CNR) for RSI C_i,N_ and conventional ADC maps. For each patient in the testing cohort (*n* = 9), CNR was estimated as the mean signal within the tumor ROI divided by the mean signal in the surrounding background tissue. Background ROIs were generated by dilating the tumor ROIs by ten pixels using the MATLAB built-in *imdilate* function with a disk-shaped morphological structuring element and subtracting the tumor mask to create a peritumoral ring representing all non-tumor tissue; in this clinical display context, bladder and bowel voxels were retained as background. In cases where the surrounding background signal was zero, a value of 1 was used as the denominator so that the metric reported the tumor mean without artificial inflation.

### 2.7. RSI Z-score maps

Z-score maps were generated to standardize RSI signal contributions C_i,N_, relative to a healthy-cervix reference. However, standard *Z*-score calculations assume that data are normally distributed, which is not the case for MRI magnitude images, which follow a Rician distribution [24]. To address this, all RSI signal contribution maps were log-transformed using a small positive offset $\left(\text{ε}=1\times 10^{-6}\right)$ to avoid log(0). After transformation, any values less than or equal to log(ε) were eliminated. Because log-transformed data may remain non-Gaussian, a robust *Z*-score estimation was performed. For each RSI compartment, C_i,N_, Z-scores were calculated using the median and median absolute deviation (MAD) of the healthy reference tissue [25]:

(2) $$Z-{\mathrm{score}}_{\text{i},\text{N}}=\frac{\text{log}\left(C_{i,N}+\varepsilon \right)-\text{median}\left[\text{log}\left(C_{i,N-\text{healthy}}+\varepsilon \right)\right]}{1.4826\times \text{MAD}\left[\text{log}\left(C_{i,N-\text{healthy}}+\varepsilon \right)\right]}$$

This approach is less sensitive to outliers and skewed distributions than standard *Z*-scores based on mean and standard deviation [26]. The reference values of $median_{i,N-\;healthy\;}$ and $MAD_{i,N-\;healthy\;}$ were estimated from cervix ROIs from the cohort of healthy volunteers (n=22) and patient RSI *Z*-maps were then expressed as Z-scores relative to this reference. A higher Z_i,N_ indicates greater compartment signal than expected in healthy cervix.

The CNR was not computed on Z-score maps because it was standardized to the healthy cervix distribution rather than to the surrounding background tissue. Instead, tumor conspicuity on Z-maps is reported directly as tumor Z-scores.

### 2.8. Statistical analysis

All statistical analyses were performed using SPSS software (version 20.0.0; IBM Corp.). Group differences in age between the model-development and independent testing patient cohorts were assessed using unpaired two-tailed *t*-tests. Menopausal status was categorized into two groups (premenopausal vs. peri/postmenopausal or declined to answer) and compared using Fisher’s exact tests.

Tumor diffusion metrics (ADC and C_i,N_) were compared between cohorts using two-tailed t-tests with Bonferroni correction for multiple comparisons (adjusted α < 0.005). The CNR was evaluated within subjects by comparing tumor C_i,N_ values against ADC using paired one-tailed *t*-tests, with Bonferroni correction. RSI *Z*-scores were also reported.

## 3. Results

**Fig. 1.**
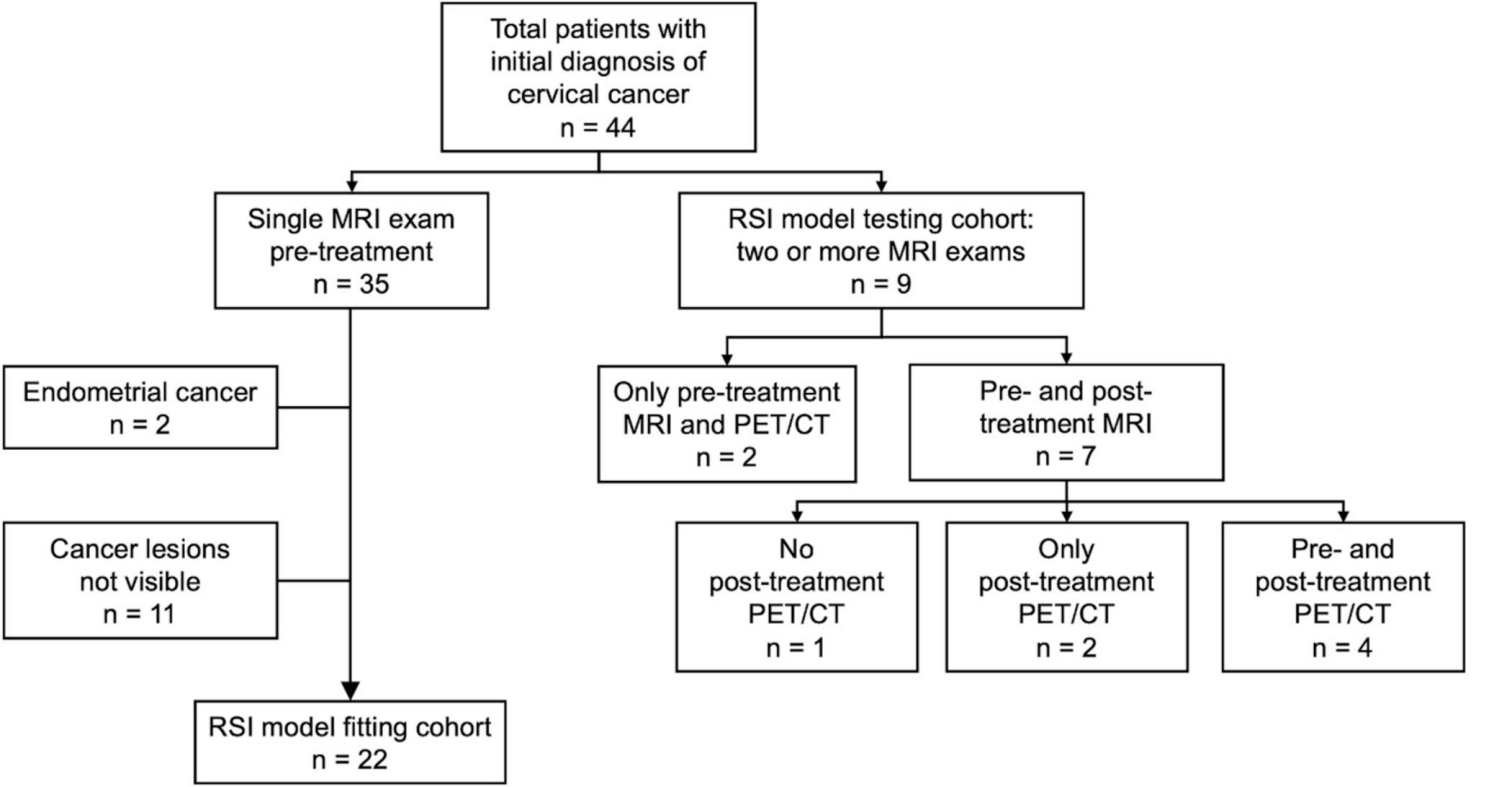
Flowchart of patient cohorts for RSI model development and independent testing. A total of 44 patients with pathology-confirmed cervical cancer were enrolled in the study. Of these, 35 underwent a single pre-treatment MRI. After excluding endometrial cancer (*n* = 2) and lesions not visible on MRI (*n* = 11), the remaining 22 patients were included in the RSI model development cohort. The independent testing cohort (*n* = 9) included patients with two or more MRI exams.

A total of 44 patients with cervical cancer were initially enrolled in the study. Of these, data from 35 patients were assigned to the RSI model development group. The remaining nine patients underwent more than one MRI examination and were used as an independent cohort for model testing (Fig. 1).

After exclusions, 22 cases remained in the model development group and nine in the testing cohort. Thirteen patients were excluded: two patients were found to have endometrial cancer (confirmed post-surgery), and in 11 patients, the tumors were too small to be observed on MRI (Fig. 1). Demographic and relevant clinical data for the patients with cervical cancer are summarized in Table 1.

There were no differences (*p* = 0.9) in menopausal status between patients with visible and non-visible tumors within the model development cohort, or between patients in the model development cohort with visible tumors and those in the independent testing cohort (*p* = 1.0).

### 3.1. RSI models for cervical cancer

**Fig. 2.**
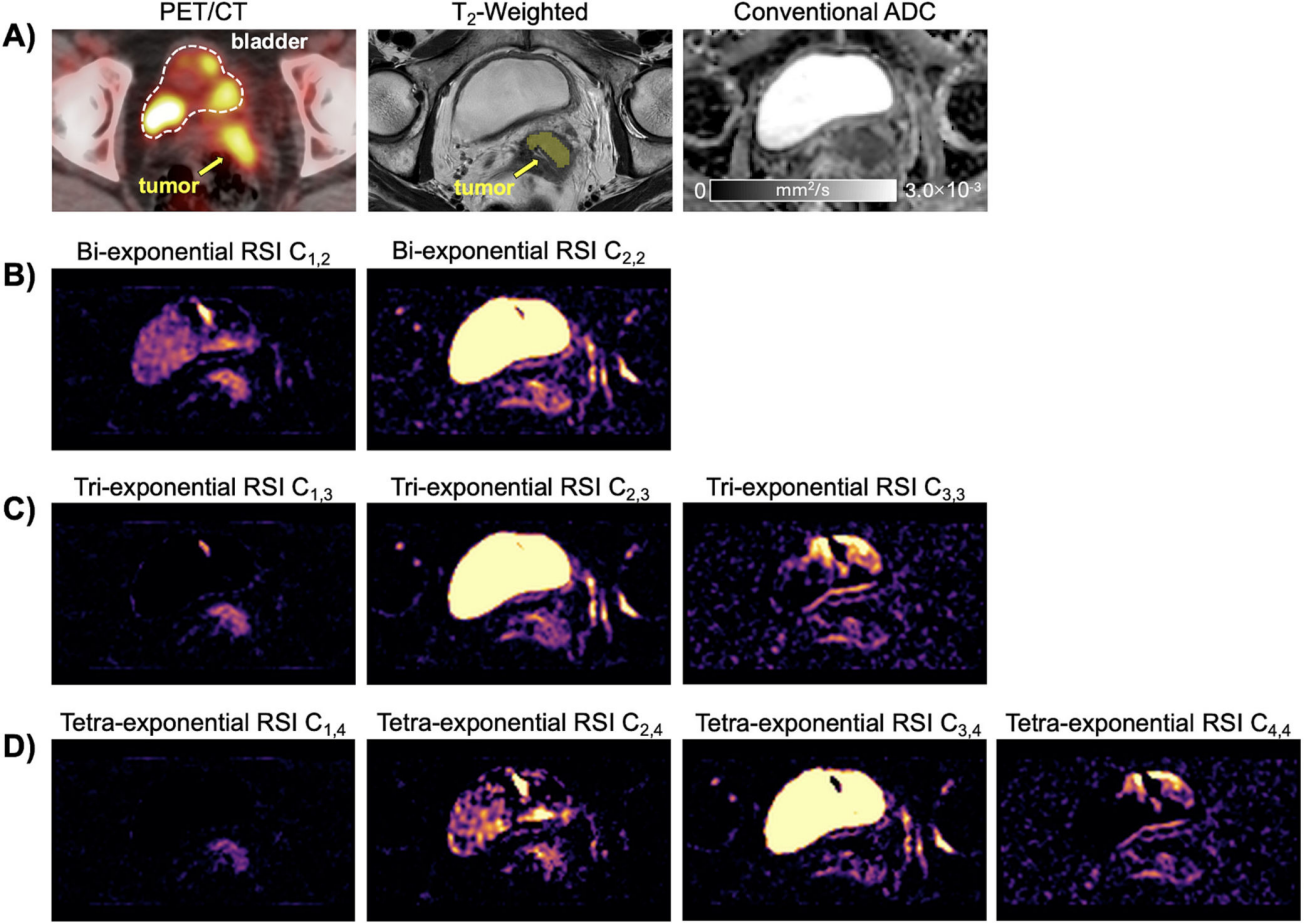
PET/CT, anatomical, and RSI signal contribution maps (C_i,N_) from a 49-year-old patient in the training cohort with FIGO stage IIB invasive squamous cell carcinoma. Tumor ROI is overlaid in yellow on the PET/CT and T₂-weighted images. A conventional ADC map computed from full-FOV DWI is also shown. RSI signal contribution maps for the bi-exponential (C_1,2_ and C_2,2_), tri-exponential (C_1,3_, C_2,3_ and C_3,3_), and tetra-exponential (C_1,4_, C_2,4_, C_3,4_ and C_4,4_) models illustrate differences in diffusion compartment representation across models and tissues. All RSI maps are displayed using the same window and level. Compartment maps demonstrate improved compartmental separation and tumor visibility with higher-order RSI models. (For interpretation of the references to colour in this figure legend, the reader is referred to the web version of this article.)

Fixed ADCs and BIC values for all multi-exponential RSI models are shown table. Signal contribution maps were generated for each C_i,N_ compartment. Among all models, the bi-exponential model had the lowest BIC value, providing the best fit to the cervical cancer diffusion-weighted signal (Table 2). The tri-exponential and tetra-exponential models increased model complexity at the cost of higher BIC values (ΔBIC = 14.1% and 6.7%, respectively). Based on the magnitude of the ADCs in this model, the slowest diffusion compartment (D_1,2_ = 1.0 × 10^−3^ mm^2^/s) likely represents a combination of restricted and hindered diffusion, while the fastest compartment (D_2,2_ = 4.5 × 10^−3^ mm^2^/s) was attributed to free fluids and bulk motion, likely reflecting vascular flow or moving urine in the bladder (Fig. 2B).

Compartment-specific signal contributions differed across models. In the bi-exponential model, tumor signal was visible in C_1,2_, however, this compartment also showed background signal in the bladder. The second compartment, C_2,2_, showed high signal in regions of free fluid and lymph nodes, whereas tumors were not visible. The tri-exponential model had improved tumor visualization, with strong signal present in C_1,3_ (and no signal in the bladder). While C_2,3_ displayed signal in lymph nodes and bladder, and C_3,3_ showed some signal in the bladder and no signal in tumors (Fig. 2C). In the tetra-exponential model, tumor signal was present in both C_1,4_ and C_2,4_, but more prominent in C_1,4_. The compartment C_3,4_ exhibited signal intensity in free fluids and C_4,4_ included signals from moving fluid in blood vessels (Fig. 2D). Notably, RSI successfully isolated Surgilube signal consistently in C_2,4_, avoiding overlap with the tumor signal in C_1,4_.

Baseline tumor diffusion measures did not differ between the RSI model-development and the independent testing cohorts (Supplemental Table 1). Median ADC (IQR) was 0.99 (0.18) × 10^−3^ mm^2^/s vs 0.89 (0.11) × 10^−3^ mm^2^/s (*p* = 0.37), respectively. Across RSI components, no differences between the cohorts were found (all *p >* 0.05). The restricted components carried the largest tumor signal in both cohorts (e.g., C_1,3_: 77.0 vs 65.5 a.u., *p* = 0.93; C_1,4_: 32.9 vs 49.0 a.u., *p* = 0.86), while hindered/free or very-fast components were comparable (C_2,4_: 70.5 vs 70.5 a.u., *p* = 0.41; C_3,4_: 13.9 vs 2.2 a.u., *p* = 0.35; C_4,4_: 8.3 vs 7.4 a.u., *p* = 0.88).

### 3.2. Tumor conspicuity

**Fig. 3.**
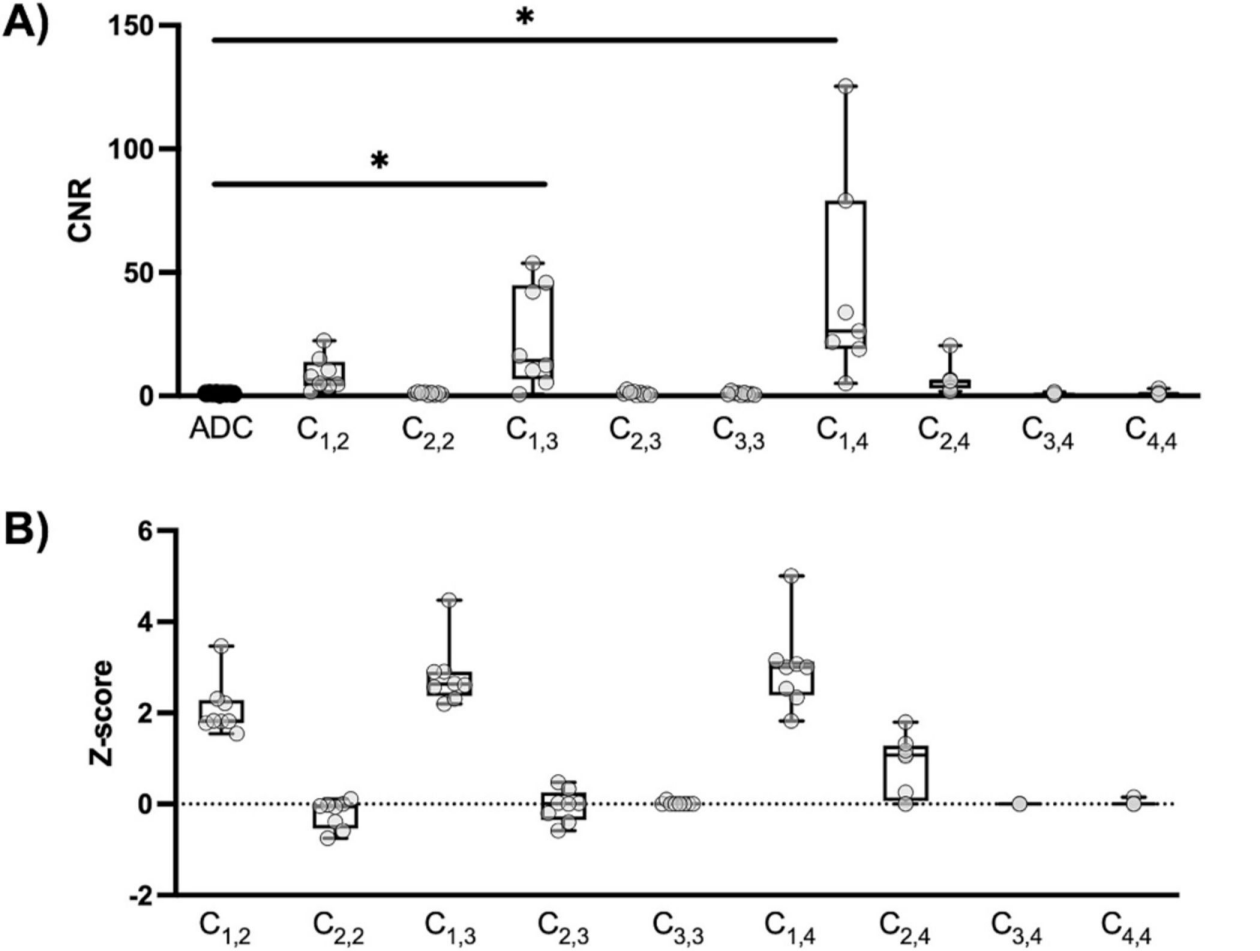
Boxplots comparing A) CNR across ADC and RSI *Z*-score maps and B) tumor *Z*-scores within RSI signal compartments (C_i,N_) in the independent testing cohort of cervical cancer patients. A) CNR was higher in RSI components C_1,3_ and C_1,4_ compared to ADC. B) Tumor Z-scores were elevated in restricted components C_1,2_ and C_1,4_, while other compartments were centered near zero. Boxes indicate interquartile range (IQR); horizontal black lines represent medians; whiskers indicate data range. Individual data points are overlaid in gray; asterisks indicate *p <* 0.05.

In the independent testing patient cohort (*n* = 9), we quantified conspicuity by computing CNR on the RSI signal contribution maps C_i,N_ and on conventional ADC. CNR was significantly higher on the restricted components of the tri- and tetra-exponential (C_1,3_ and C_1_,_4_) than on ADC (Fig. 3A). Median (IQR) CNR was 12.5 (36.7) for C_1,3_ and 21.8 (28.8) for C_1,4_. Overall, RSI signal contribution maps across these two RSI models displayed improved visual tumor conspicuity with increasing model complexity (Fig. 3A).

### 3.3. RSI Z-score maps

Participants in the healthy cohort had a median age of 45 years (IQR: 9.8 years, range: 22–63 years) and self-reported menopausal status as pre-menopausal (*n* = 12), perimenopausal (*n* = 3), postmenopausal (*n* = 6), or declined to answer (n = 1). RSI compartment maps (C_i,N_) from the reference group of healthy volunteers were used to calculate the median (median_1_,_N-healthy_) and median absolute deviation (MAD_i_,_N-healthy_) for each compartment (Supplemental Table 2). These reference values were then used to generate *Z*-score maps of the corresponding C_i,N_ maps from cervical cancer patients in the independent testing cohort.

**Fig. 4.**
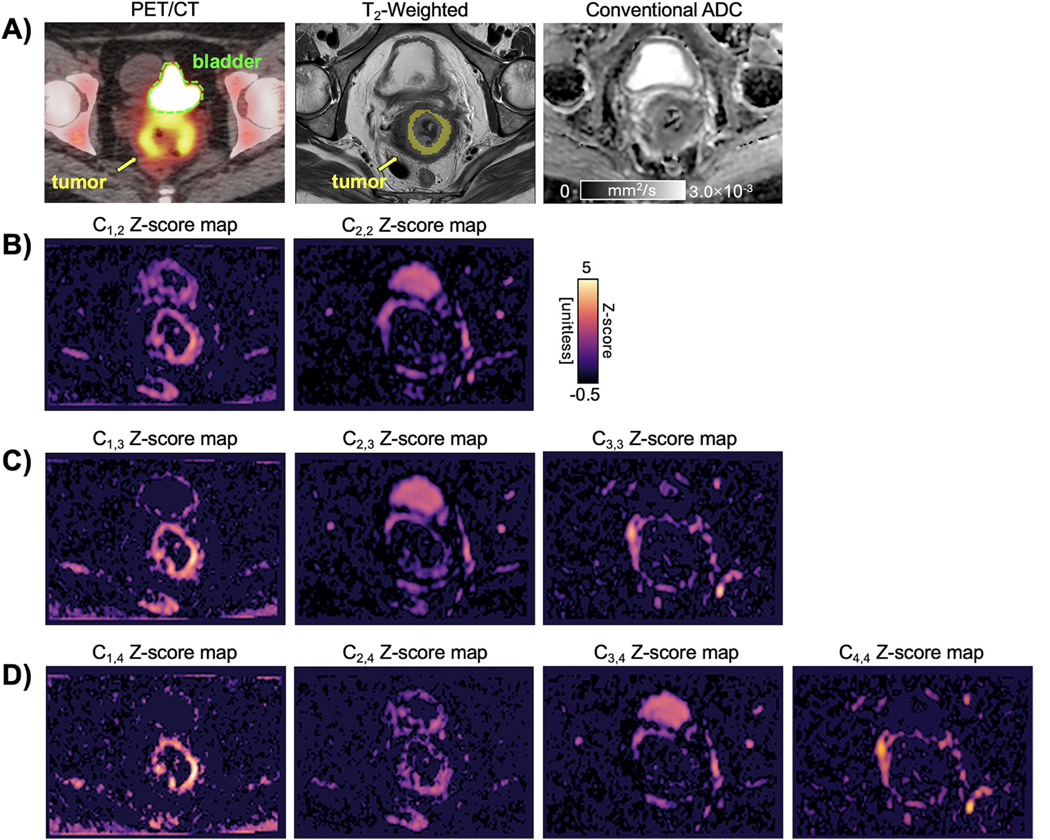
PET/CT, anatomical, and RSI signal contribution maps (C_i,N_) from a 36-year-old patient in the testing cohort with FIGO stage IIIC invasive squamous cell carcinoma. Tumor ROI is overlaid in yellow on the PET/CT and T₂-weighted images. A conventional ADC map is also shown. *Z*-score maps for each RSI compartment were generated by comparing voxel-wise log-transformed signal contributions to the mean and standard deviation from healthy volunteer data. Z-score maps for the bi-exponential (C_1,2_ and C_2,2_), tri-exponential (C_1,3_, C_2,3_ and C_3,3_), and tetra-exponential (C_1,4_, C_2,4_, C_3,4_ and C_4,4_) models indicate the deviation in signal compared to healthy volunteers. All RSI *Z*-score maps are displayed using the same window and level. (For interpretation of the references to colour in this figure legend, the reader is referred to the web version of this article.)

We found that Z-scores in tumor lesions were highest in compartments C_1,3_, and C_1,4_ (Fig. 3B), indicating marked deviation from normal restricted water compartments in tumor tissue. Median (IQR) Z-scores of compartments C_1,2_, C_1,3_ and C_1,4_ were 1.7 (0.5), 2.4 (0.4), and 2.7 (0.6), respectively. In contrast, the *Z*-scores in all other compartments remained near zero, consistent with their limited contribution to tumor signal. As shown in Fig. 4, tumor signal is primarily contained in C_1,N_ compartments, which is consistent with the near-zero *Z*-scores observed in healthy tissue for these compartments.

BIC analysis showed that higher-order models did not substantially overfit the data, with ΔBIC increases of 14.1% and 6.7% for the tri- and tetra-exponential models relative to the bi-exponential model. The tetra-exponential model had the highest CNR and was therefore selected as the optimal model for cervical cancer.

### 3.4. Longitudinal observations

**Fig. 5.**
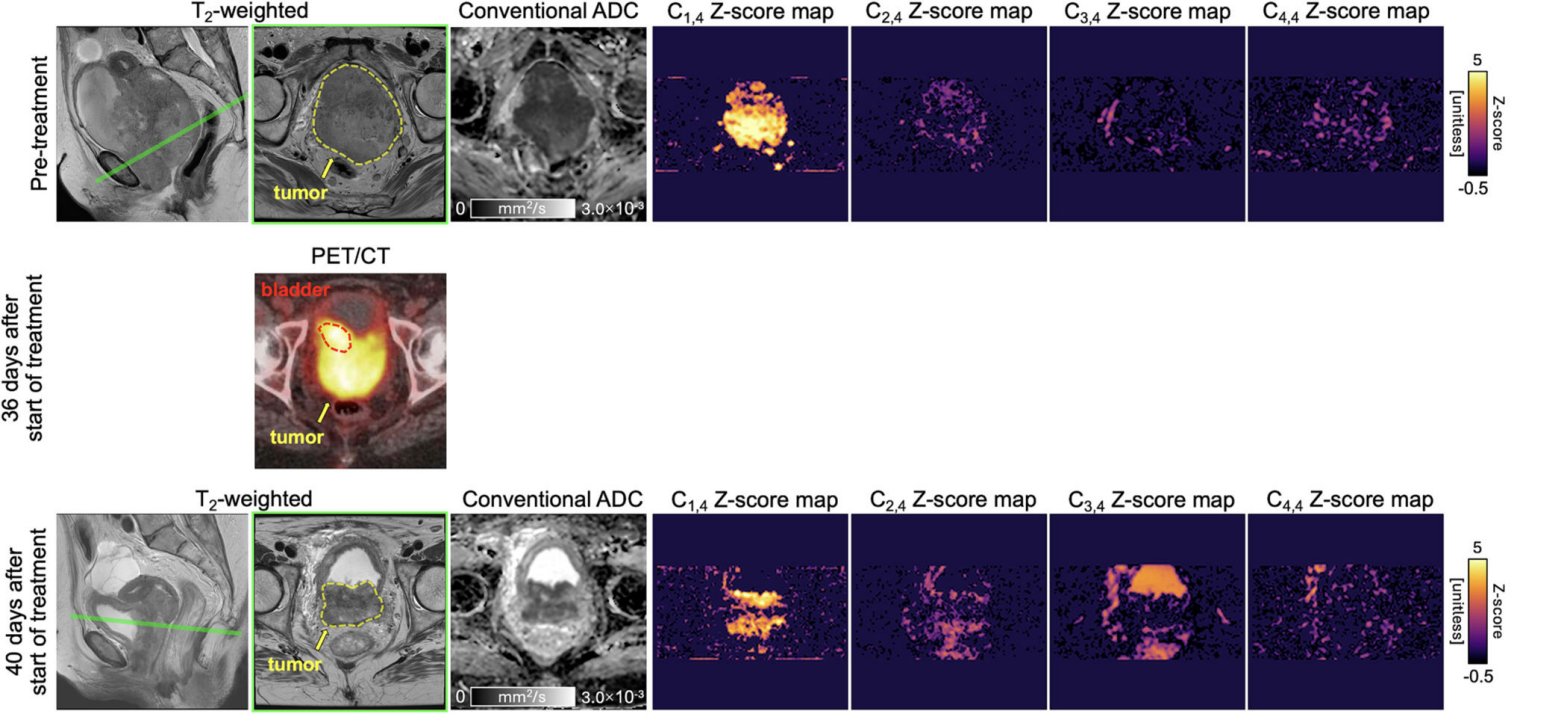
Longitudinal imaging for a 62-year-old patient in the testing cohort with FIGO stage IV invasive poorly differentiated squamous cell carcinoma. Sagittal and oblique axial T_2_-weighted, conventional ADC maps, and voxel-wise Z-score maps from the tetra-exponential RSI model (C_i,4_) before and 40 days after the beginning of treatment are shown. All Z-score maps are displayed using the same window and level. Yellow dashed contour indicates the tumor region in MR images. Green lines on sagittal images indicate the prescription of the oblique axial images. PET/CT of a similar anatomical location is show for PET/CT 36 days after the beginning of treatment. Red dashed contour indicates the location of the bladder. (For interpretation of the references to colour in this figure legend, the reader is referred to the web version of this article.)

Eight patients underwent more than one MRI during the study period. Because timing and treatment status varied owing to clinical management, these data were underpowered for post-treatment analyses. However, we present illustrative cases to show RSI *Z*-map characteristics in longitudinal scans. A single patient had residual cancer at the time of the second MRI exam (Fig. 5). For this patient, C_1,4_ *Z*-score decreased from a pre-treatment median of 2.2 (IQR: 3.2) to a post-treatment median of 1.9 (IQR: 2.9).

**Fig. 6.**
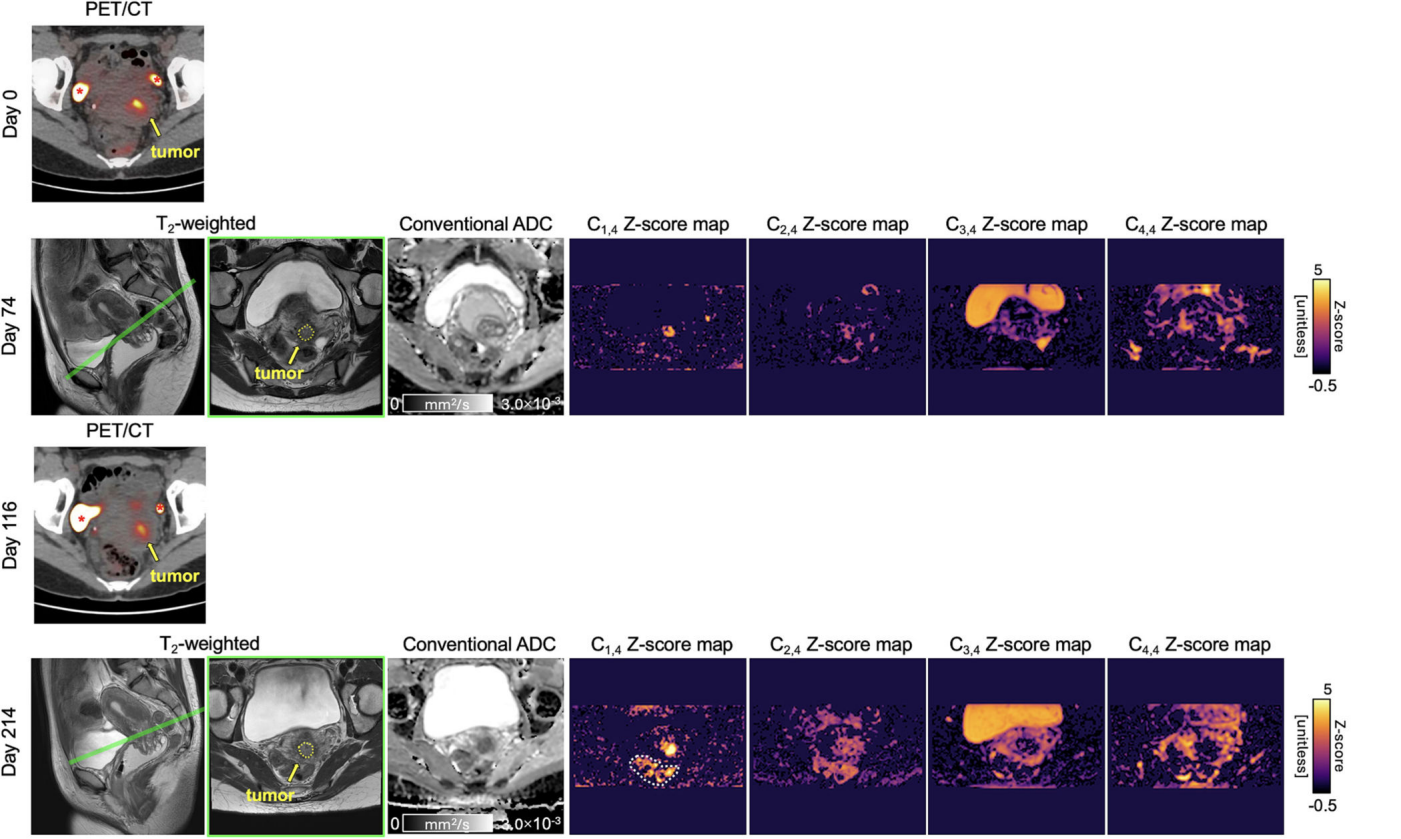
Longitudinal imaging for a 37-year-old patient in the testing cohort with adenocarcinoma in-situ FIGO stage IB2 cervical cancer. PET/CT was acquired at days 0 and 116, while MRI was performed at days 74 and 214 after the initial PET/CT. Sagittal and oblique axial T_2_-weighted, conventional ADC maps, and voxel-wise *Z*-score maps from the tetra-exponential RSI model (C_i,4_). All Z-score maps are displayed using the same window and level. Yellow dashed contour indicates the tumor region in MR images. Green lines on sagittal images indicate the prescription of the oblique axial images. Red asterisks in PET/CT images indicates the location of the bladder. (For interpretation of the references to colour in this figure legend, the reader is referred to the web version of this article.)

**Fig. 7.**
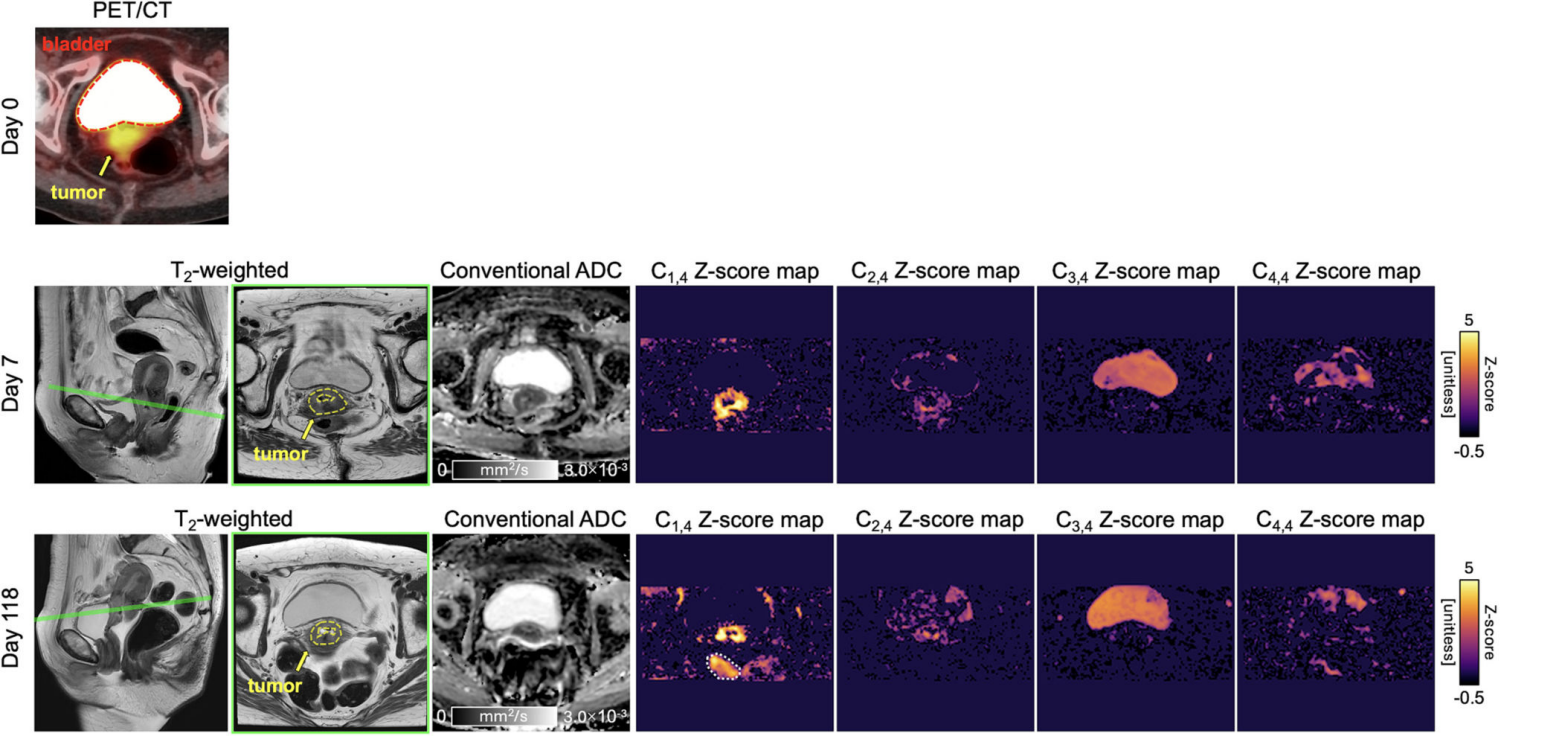
Longitudinal imaging acquired prior to treatment in a 70-year-old patient in the testing cohort with FIGO stage IIB invasive squamous cell carcinoma. Imaging timepoints include PET/CT and multiparametric MRI 7 and 118 days after the PET/CT. Sagittal and oblique axial T_2_-weighted, conventional ADC maps, and voxel-wise Z-score maps from the tetra-exponential RSI model (C_i,4_). All Z-score maps are displayed using the same window and level. Yellow dashed contour indicates the tumor region in MR images. Green lines on sagittal images indicate the prescription of the oblique axial images. Red dashed contour in PET/CT images indicates the location of the bladder. (For interpretation of the references to colour in this figure legend, the reader is referred to the web version of this article.)

Longitudinal imaging in two patients with serial MRI exams and no intervening treatment is shown in Figs. 6 and 7. One patient elected not to initiate therapy during this study and underwent two MRI scans. A second patient completed two pre-treatment exams before continuing care elsewhere. RSI *Z*-score maps showed persistently elevated signal within cervical cancer in the C_1,4_ compartment across time points (yellow dashed contours). Areas of fecal material in the rectum also demonstrated elevated C_1,4_ signal representing a source of false-positive findings (white dashed contours). In the first patient, C_1,4_ Z-scores within the tumor were similar (medians 2.0 with IQR 1.8, and 2.1 with IQR 3.4, Fig. 6). The second patient had median C_1,4_ Z-scores of 3.4 (IQR: 1.8) at the first timepoint and 3.5 (IQR: 2.3) at the second (Fig. 7). Focal C_1,4_ signal in bowel contents was occasionally observed and is noted as a potential false positive.

## 4. Discussion and conclusions

This study demonstrates the clinical potential of RSI applied to cervical cancer while incorporating *Z*-score maps to standardize RSI metrics against healthy tissue. Our results demonstrate that RSI-derived maps have higher tumor conspicuity compared to conventional ADC, particularly when using tri- and tetra-exponential models. These findings support the feasibility of RSI in improving lesion visualization and may enable quantitative assessment of cervical tumors.

Among the RSI models evaluated, the bi-exponential model had the lowest BIC, indicating the best fit to the cervical cancer diffusion signal. However, CNR measurements showed improved tumor conspicuity with model complexity. The tetra-exponential model provided the highest CNR, with only a 6.7% increase in BIC relative to the bi-exponential model, indicating no substantial overfitting. Therefore, the tetra-exponential model was the most suitable for cervical cancer, as it balances model fit with the practical outcome of improved lesion visualization. Z-score analyses further supported this conclusion. Z-scores were elevated primarily in C_1,N_ compartments, with tumor signal concentrated in C_1,3_ and C_1,4_. In contrast, Z-scores for other compartments remained near zero, consistent with minimal tumor contribution. These findings suggest that deviations in C_1,N_ compartments reflect abnormal restricted diffusion in cervical cancer, while other compartments capture background or non-tumor tissues.

The cervical cancer-specific RSI tetra-exponential model included compartments corresponding to hindered, free, and flow-related components, respectively. We interpret the restricted compartment, C_1,4_, as indicating that restricted diffusion in highly cellular cervical tumors, where reduced extracellular space limit water mobility. The intermediate and faster compartments may reflect contributions from extracellular space, interstitial fluid, and vascular flow, which together contribute to the measured diffusion signal in tumors. These compartments link the RSI signal to underlying tumor microstructure.

In other cancer types, RSI models have used similar multi-compartment frameworks with variations in model parameters reflecting differences in underlying tissue microstructure [16,24]. For example, in a study evaluating prostate cancer bone metastases using whole-body diffusion-weighted MRI, a four-compartment model included a zero-valued component (D_1,4_ = 0 mm^2^/s) together with higher coefficients of 1.1 × 10^−3^ (D_2,4_), 2.8 × 10^−3^ (D_3,4_), and *>* 3.0 × 10^−2^ (D_4,4_) mm^2^/s [27]. A similar approach was used in breast cancer, where the RSI tri-exponential model used fixed ADCs of 0 (D_1,3_), 1.5 × 10^−3^ (D_2,3_), and 10.8 × 10^−3^ (D_3,3_) mm^2^/s [17]. These zero-valued RSI ADCs have been previously interpreted as the component of the diffusion-weighted signal that causes the signal to persist at high b-values in tumors, including slow or restricted diffusion and lengthened T_2_ relaxation time [8]. These tissue-specific properties support the need for developing organ-specific RSI models.

Unlike conventional ADC estimation, which divides the diffusion-weighted signal by S₀ to isolate diffusion effects [15], RSI models the diffusion-weighted signal without this normalization, thereby preserving T_2_ weighting and other signal contributions embedded in the data [8]. We hypothesize that retaining these components, particularly T_2_ weighting, contributes to improved tumor visualization by preserving intrinsic tissue contrast. However, this also means that RSI outputs are expressed in arbitrary units of image intensity. To address this, *Z*-score normalization transforms C_i,N_ values into unitless standardized deviations based on healthy reference distributions, thus improving reproducibility across patients [21]. The implementation of cervical cancer RSI across vendors requires reference distributions to be generated separately for each vendor. Future work may evaluate the feasibility of normalization to a shared reference is feasible and whether this allows for consistent interpretation of RSI Z-score maps across vendors. Studies in prostate cancer show promising results for RSI quantitative biomarkers across vendors, protocols, and centers [19,20].

We observed that fecal matter in the rectum can exhibit an elevated signal in the C_1,4_ compartment, potentially contributing to false-positive findings. Signal-confounds from retained stool can be reduced by using mini-enemas before the MR exam [28–30]. While mini-enemas can help minimize signal from fecal material and artifacts from gas, they do not address bowel motion artifacts, which remain a source of image distortion in pelvic MRI. To mitigate these effects, antiperistaltic agents (e.g., glucagon or butylscopolamine) are often administered [30,31]. In addition to bowel-related signals, lymph nodes may display elevated signals in C_1,4_, presenting another potential confound. It is currently unclear how to best distinguish benign from malignant lymph node findings with RSI. Further model development is needed to improve the specificity of RSI in evaluating lymph node status.

Conventional ADC maps, while widely available and easy to compute, remain useful in the clinical staging of cervical cancer by helping define tumor extent, parametrial invasion, and involvement of adjacent structures [32]. Our results demonstrate that ADC maps have lower tumor conspicuity compared to RSI-based metrics. This is consistent with prior reports showing that ADC suffers from low specificity and can be confounded by post-treatment changes [33–35], limiting its utility for individual-level decision-making. However, both conventional ADC and RSI remain vulnerable to image distortion and signal loss caused by B_0_ inhomogeneities, particularly in the pelvis where bowel gas is common [32,36]. These susceptibility artifacts continue to pose challenges for diffusion imaging in this anatomical region and must be considered when evaluating image quality or interpretability. There is a need for diffusion techniques that are both robust to artifacts and capable of capturing microstructural details.

While DCE is widely used for diagnosis, staging, and treatment monitoring across many cancer types, its utility in cervical cancer is limited [32]. DCE metrics have shown low specificity in differentiating residual or recurrent tumor from post-treatment inflammation and fibrosis, limiting their utility in post-treatment assessment of cervical cancer [37,38]. In contrast, diffusion-based approaches such as RSI are contrast-free and less sensitive to perfusion-related confounds. DWI-based techniques may also avoid the use of intravenous contrast agents, which have been associated with gadolinium deposition in the brain and other tissues after repeated imaging [39]. Although DCE provides higher spatial resolution than DWI, emerging acceleration techniques have shown promise in enabling high-resolution DWI in the pelvis [40,41]. In future work, we will evaluate the application of a high-resolution DWI pulse sequence, which has shown promise in detecting smaller lesions in breast cancer, to determine whether it may improve detection sensitivity in cervical cancer.

This study had several limitations. Owing to the limited cohort size, we were unable to formally evaluate the sensitivity or specificity of RSI in staging or evaluating cervical cancer. Second, all imaging was acquired on a single MRI vendor, limiting the generalizability of the RSI *Z*-score standardization. Future studies will validate these findings in larger, multi-institutional cohorts and investigate whether RSI Z-scores correlate with clinical outcomes such as treatment response.

Our study demonstrates that a cervical cancer-specific RSI framework using a tetra-exponential model improves tumor conspicuity compared with conventional ADC. Z-score mapping further standardized compartment signals relative to healthy cervical tissue, improving interpretability and reproducibility. Together, these approaches support the feasibility of RSI as a contrast-free imaging tool for lesion evaluation in cervical cancer.

## Supplementary Material

Supplemental Table 1. Baseline tumor diffusion metrics by patient cohort

## Figures and Tables

**Table 1:** Demographic data of cervical cancer patients in the study. Median values are reported for age and tumor size. Values in parenthesis are the range.

	RSI model development		Independent testing cohort
Tumor visible on MRI	Y	N	Y
**No. of patients**	22	11	9
**Age (years)**	42 (29–82)	45 (29–69)	46 (36–70)
**Menopausal Status**			
Premenopausal	9	6	4
Perimenopausal		1	2
Postmenopausal	8	4	3
Declined to answer	5		
**Tumor size (cm×cm×cm)**	3.4 × 3.3 × 4.0 (1.5–10, 0.8–6.7, 1.3–7.5)	Not reported	3.8 × 2.8 × 3.4 (2.3–20, 2.0–7.1, 3.1–7.0)
**Histologic type**			
Adenocarcinoma	5	4	1
Adenosquamous cell carcinoma			1
Squamous cell carcinoma	17	7	7
**Staging**			
I		1	
IA1	1	2	
IA2	1	2	
IB		1	
IB1	1	2	1
IB2	3	1	3
IB3	1		
IIB	8		1
IIIB	3		1
IIIC			1
IIIC1	2		
IIIC2			1
IVA	2		
IVB		1	1
N/A		1	
**Treatment**			
Chemoradiotherapy	19	3	8
Surgery None	3	8	1

**Table 2:** Estimated organ-specific RSI diffusion coefficients D_i,N_ for bi-, tri- and tetra-exponential models and the relative Bayesian information criterion (BIC) for all models.

RSI model	RSI Parameter	Using cancer voxels included only	ΔBIC (%)
	D_1,2_	1.0 × 10^−3^ mm^2^/s	
Bi-exp	D_2,2_	4.5 × 10^−3^ mm^2^/s	–
	D_1,3_	0.9 × 10^−3^ mm^2^/s	
Tri-exp	D_2,3_	2.9 × 10^−3^ mm^2^/s	14.1%
	D_3,3_	62.2 × 10^−3^ mm^2^/s	
	D_1,4_	0.7 × 10^−3^ mm^2^/s	
	D_2,4_	1.9 × 10^−3^ mm^2^/s	
Tetra-exp	D_3,4_	3.9 × 10^−3^ mm^2^/s	6.7%
	D_4,4_	641.0 × 10^−3^ mm^2^/s	
			

## Data Availability

Summary data available upon reasonable request to the corresponding author.

## Appendix A. Supplementary data

Supplementary data to this article can be found online at 10.1016/j.mri.2026.110692.
