# Supplementary material for: Development and evaluation of a cervical cancer-specific restriction spectrum imaging (RSI) model
Source: Magn Reson Imaging. Author manuscript; Available in PMC 2026 Jul 14. (PMC13367980; doi:10.1016/j.mri.2026.110692)
Supplement: Supplemental Table 1. Baseline tumor diffusion metrics by patient cohort [file NIHMS2180367-supplement-Supplemental_Table_1__Baseline_tumor_diffusion_metrics_by_patient_cohort.docx]

**Supplemental Table 1.** Baseline tumor diffusion metrics by patient cohort. Median and IQR values for tumors in the RSI model-development and independent testing cohorts. ADC (×10^-3^ mm^2^/s) was computed from full-FOV clinical DWI and C_i,N_ in arbitrary units (a.u.) were estimated from reduced-FOV multi-shell DWI. Reported values are the median (interquartile range, IQR)

| Parameter | RSI model  development cohort (n=22) | Testing cohort  (n=9) | p-value |
| --- | --- | --- | --- |
| ADC [×10^-3^ mm^2^/s] | 0.99 (0.18) | 0.89 (0.11) | 0.07 |
| C_1,2_ [a.u.] | 108.0 (98.3) | 71.8 (37.0) | 0.77 |
| C_2,2_ [a.u.] | 79.8 (44.0) | 63.5 (35.3) | 0.17 |
| C_1,3_ [a.u.] | 77.0 (87.8) | 65.5 (30.4) | 0.93 |
| C_2,3_ [a.u.] | 73.2 (48.4) | 61.7 (50.9) | 0.23 |
| C_3,3_ [a.u.] | 9.7 (17.5) | 9.6 (7.2) | 0.89 |
| C_1,4_ [a.u.] | 32.9 (62.1) | 49.0 (23.7) | 0.86 |
| C_2,4_ [a.u.] | 70.5 (66.8) | 70.5 (51.8) | 0.41 |
| C_3,4_ [a.u.] | 13.9 (26.1) | 2.2 (4.1) | 0.35 |
| C_4,4_ [a.u.] | 8.3 (17.2) | 7.4 (4.6) | 0.88 |

**Supplemental Table 2.** Median (median_i,4-healthy_​) and standard deviation (MAD_i,4-healthy_) of log-transformed RSI signal contribution compartments (C_i,N_) in healthy cervix tissue. These values were derived from 11 healthy volunteers and used to generate RSI C_i,N_​ Z-score maps. a.u. = arbitrary units.

| RSI Signal Contribution Compartment | Healthy Cervix | |
| --- | --- | --- |
|  | Median (median_i,N-healthy_​; a.u.) | Median Absolute Deviation  (MAD_i,N-healthy_; a.u.) |
| C_1,2_ | 2.9 | 0.8 |
| C_2,2_ | 4.4 | 0.7 |
| C_1,3_ | 2.4 | 0.8 |
| C_2,3_ | 4.2 | 0.7 |
| C_3,3_ | 3.6 | 0.7 |
| C_1,4_ | 2.0 | 0.7 |
| C_2,4_ | 3.5 | 0.8 |
| C_3,4_ | 4.1 | 0.7 |
| C_4,4_ | 3.5 | 0.7 |
